# Oral health in China: from vision to action

**DOI:** 10.1038/s41368-017-0006-6

**Published:** 2018-01-17

**Authors:** Xuedong Zhou, Xin Xu, Jiyao Li, Deyu Hu, Tao Hu, Wei Yin, Yujiang Fan, Xingdong Zhang

**Affiliations:** 1 State Key Laboratory of Oral Diseases & National Clinical Research Center for Oral Diseases & Department of Operative Dentistry and Endodontics, West China Hospital of Stomatology, Sichuan University, Chengdu, China; 2 State Key Laboratory of Oral Diseases & National Clinical Research Center for Oral Diseases & Department of Preventive Dentistry, West China Hospital of Stomatology, Sichuan University, Chengdu, China; 3 National Engineering Research Center for Biomaterials, Sichuan University, Chengdu, China

## Abstract

Chinese president Xi Jinping made clear at the National Health and Wellness Conference that health is the prerequisite for people’s all-around development and a precondition for the sustainable development of China. Oral health is an indispensable component of overall health in humans. However, the long neglect of oral health in overall health agendas has made oral diseases an increasing concern. With this perspective, we described the global challenges of oral diseases, with an emphasis on the challenges faced by China. We also described and analyzed the recently released health policies of the Chinese government, which aim to guide mid-term and long-term oral health promotion in China. More importantly, we called for specific actions to fulfill the larger goal of oral health for the nation. The implementation of primordial prevention efforts against oral diseases, the integration of oral health into the promotion of overall health, and the management of oral diseases in conjunction with other chronic non-communicable diseases with shared risk factors were highly recommended. In addition, we suggested the reform of standard clinical residency training, the development of domestic manufacturing of dental equipment and materials, the revitalization traditional Chinese medicine for the prevention and treatment of oral diseases, and integration of oral health promotion into the Belt and Road Initiative. We look forward to seeing a joint effort from all aspects of the society to fulfill the goal of Healthy China 2030 and ensure the oral health of the nation.

## Placing dental care at the center of health policy

Government initiatives should promote high quality dental care to improve the overall health of the population, say Chinese researchers. Oral infections cost the global economy millions of dollars annually, and are closely associated with diseases such as diabetes and cancer. Improving oral health is a central goal of the Healthy China 2030 program, launched recently by the Chinese government. Xuedong Zhou at Sichuan University in Chengdu and co-workers reviewed challenges facing oral health in China and suggest guidelines to enable the government to achieve their aims. Tooth decay and gum disease are key concerns, and recent policy changes emphasize the importance of healthy lifestyles, integrating oral health into regular physical checks, and educating the population. The researchers also recommend managing oral health during treatment for other chronic diseases, and ensuring high quality training for professionals.

## Introduction

The Chinese president Xi Jinping made a keynote speech at the National Health and Wellness Conference on 19th August 2016. The speech made clear that health is the prerequisite for people’s all-around development and a precondition for the economic and social development of China^1^. The Chinese government is determined to give strategic priority to developing people’s fitness and accelerating the development of a healthy China. This speech set a clear direction for China’s health policy in the future. Since October 2016, the Chinese government has released a series of policies regarding health and healthy development in China. The Healthy China 2030 blueprint released by the Central Committee of the Communist Party of China in October 2016 underlined five specific targets for the all-around development of healthy China in the next 15 years, which includes improving the country’s health level, controlling major risk factors, increasing the health service capacity, expanding the scale of health industry, and optimization the health service system^2^. Of note, most of the health policies released by the government cover the promotion of oral health, indicating the tremendous political will of the Chinese government to invest in public oral health. The central idea of these policies is to realize a paradigm shift from a treatment-centered practice to the prevention-oriented management of oral diseases. All these policies will guide mid-term to long-term efforts toward oral health promotion and provide good opportunities for the development of sustainable public oral health in China.

## The challenge of oral diseases

Chronic non-communicable diseases (NCDs) have become a global concern. According to the WHO’s Global Status Report on non-communicable diseases 2014^3^, NCDs are becoming the major cause of global mortality. The rapid increase in the incidence of chronic diseases and the associated mortality and medical expenses have become a heavy burden on society. Most oral diseases are NCDs. The number of people with untreated oral conditions worldwide increased from 2.5 million in 1990 to 3.5 billion in 2015, with a 64% increase in disability-adjusted life years (DALYs) due to oral conditions^4^.

The most common oral diseases include untreated caries in permanent teeth, untreated caries in deciduous teeth, and severe periodontitis in adults. The global age-standardized prevalence rates of these oral diseases in 2015 were 34.1%, 7.8%, and 7.4%, respectively^4^. Among 100 diseases that affect DALYs, severe periodontitis, untreated dental caries and missing teeth rank in the 77th, 80th, and 81st place, respectively. These three diseases were responsible for the loss of 224 healthy years in every 100 thousand people on average^5^. Updated data from the Global Burden of Diseases, Injuries, and Risk Factors Study (GBD) 2016, as recently released by the journal *Lancet*, showed that worldwide, dental caries in permanent teeth had the highest prevalence (2.44 billion, 95% UI 2.29 billion to 2.59 billion) of all diseases afflicting human beings. In addition, the global incidence of caries in permanent teeth (7.26 billion, 6.72 billion to 7.84 billion) and caries in deciduous teeth (1.76 billion, 1.26 billion to 2.39 billion) ranked 2nd and 5th, respectively, among the ten diseases with the highest incidence in 2016^6^. Oral diseases were also the 4th highest cause of financial burden from diseases in most industrial countries. The direct treatment costs for dental diseases worldwide were estimated to be 298 billion USD per year, corresponding to an average of 4.6% of the global health expenditure. The estimated indirect costs of dental diseases worldwide was 144 billion USD per year, corresponding to economic losses within the range of the 10 most frequent global causes of death (between 895 billion USD for cancer and 126 billion USD for lower respiratory infections)^7^. In the US, the total cost of oral diseases reached 122 billion dollars in 2014^8^. Medical cost has become a serious problem for global economic development. More importantly, oral infectious diseases, particularly periodontitis, are also closely associated with systemic diseases such as diabetes, cardiovascular diseases, rheumatoid arthritis, preterm birth, respiratory diseases, colorectal cancer, inflammatory bowel diseases and Alzheimer’s disease^9–19^. Because of its shared risk factors and its two-way relationship with some systemic diseases, oral diseases are receiving global attention from health care professionals, governments, and insurance and pharmaceutical companies.

According to recently released data obtained from the 4th national oral health epidemiology survey^20^, dental caries and periodontal disease are still major diseases that affect the oral health of Chinese people. They are also the main causes of missing teeth in the middle-aged and elderly populations. Specifically, the reported caries prevalence rates of children aged 5 years and 12 years were 70.1% and 34.5%, respectively, relatively higher than the caries prevalence rates reported by the 3rd national oral health epidemiology survey in 2005. Although the tooth filling ratio increased by approximately 50% compared with the data reported 12 years ago, it is still relatively low compared with developed countries. In addition, the rate of periodontal health is 12.6% in middle-aged and elderly people, even lower relative to the data reported 12 years ago (approximately 22.7% of population were periodontally healthy according to the 3rd national oral health epidemiology survey). Clearly, oral diseases are still highly prevalent in China. However, specialized dental care is not generally available, and it is often unaffordable for many people due to limited insurance coverage, posing a serious public health challenge to policy makers. China has been experiencing a huge change since the government realized the critical problem of human health, including oral health. Hence, the Chinese government has recently released a series of health policies with a particular focus on promoting oral health.

## Current oral health policies in China

The Central Committee of the Communist Party of China and the State Council of China released the Healthy China 2030 blueprint in October 2016. It included 29 chapters stating that China will advocate healthy lifestyles, improve health services, optimize health industries and build a medical system that provides basic health to every citizen by 2020. China will continue to improve its health sectors, and main health indicators are expected to reach the standards of developed countries by 2030^2^.

To implement the 2030 Health Plan, the State Council of China released the 13th five-year plan for health in December 2016^21^. This plan is a major part of the 13th five-year plan for national economic and social development. According to the 13th five-year plan for health, the major tasks of oral health promotion from 2016–2020 will focus on (1) including oral health examination as a regular part of the conventional physical examination, (2) integrating intervention for populations susceptible to oral diseases into the comprehensive program for the prevention of chronic diseases, (3) advocating healthy lifestyles, including reductions in salt, fat and sugar consumption, and promoting better management of oral health, body weight and musculoskeletal health, and (4) speeding up the development of oral health-related industries to satisfy people’s increasing oral health demands.

The general office of the National Health and Family Planning Commission (NHFPC) printed and distributed the Regulation of the National Demonstration Area for Comprehensive Prevention and Control of Chronic and Non-Communicable Disease on October 20, 2016^22^. These regulations require that oral health education be provided in all primary schools and kindergartens and that suitable measures, such as topical fluoridation and pit and fissure sealing, should be administered to children and other high-risk groups.

The General Office of State Council printed and distributed the National Program for Chronic Disease Control and Prevention (2017–2025) (Chronic Diseases Program), another document for implementing the Healthy China 2030 Plan, on January 22, 2017^23^. This program underlines 5 major chronic diseases that require specific preventive efforts, i.e., cardiovascular diseases, malignant tumors, diabetes, chronic obstructive lung diseases and oral diseases. Strategies related to oral health, as proposed by the Chronic Diseases Program, include (1) promoting oral health education in preschool, primary school and middle school; (2) developing techniques and supportive instruments to help people maintain their own oral health; (3) promoting early intervention among urban and rural residents through the efforts of community health service centers and township hospitals; (4) including oral health examinations as a regular part of conventional physical examinations; (5) developing personalized interventions for children and the elderly, with a focus on the management of dental caries and periodontal diseases; (6) implementing topical fluoridation and pit and fissure sealing and other oral health care measures to reduce the caries prevalence rate to below 30% in 2025.

These policies, regulations and guidelines serve as a cornerstone of the Chinese government’s efforts by recognizing oral health as an important part of overall health and integrating it into the mid-term and long-term programs for the management of NCDs.

## From vision to action

### Government as the first barrier against oral diseases

The government’s function as the first barrier against diseases is an “upstream prevention” strategy for addressing chronic NCDs. The government can prevent the harmful aspects of NCDs by establishing laws, regulations and guidelines that result in the good implementation of primordial prevention that avoids the development of risk factors in the first place. Upstream prevention, which targets risk factors to prevent diseases, is different from the traditional primary prevention included in the “prevention, treatment, and rehabilitation” paradigm. The government-guided upstream prevention targets risk factors before the onset of disease by addressing society, economics, education, the environment, politics, human behaviors and other non-medical factors. For example, the government can make policies that encourage factories to produce more sugar-free diet drinks and xylitol-containing products, thus reducing the sugar intake of the population and consequently preventing dental caries^24^. Another good example is the development of a healthy city, which has been promoted by WHO since 1986^25^. The healthy city concept represents a paradigm shift from a treatment-centered health care system to the one that combines treatment, prevention, and the promotion of health policy. The active participation of the local government to guide the transfer of responsibility from health professionals to the entire society is a particular emphasis of the health city project^26^. The healthy city project could ultimately benefit overall human health, including oral health.

The health policies recently released in China underline the important role of the government in the management of NCDs, including oral diseases. The release of these policies marks the first time that oral health promotion has been addressed in such high-level documents, reflecting the strong determination of the Chinese government on this issue. These oral health-related policies can guide the activities of government at all levels, promoting collaboration between related departments, the reconciliation of related interests, and the establishment of an oral health evaluation system, a multilateral cooperation system and a compensatory system for policy losses. All levels of government should act with a clear sense of duty and a specific focus and should place joint efforts on the prevention of harmful factors related to overall health from all aspects of life. This preemptive upstream prevention strategy could ultimately constitute an effective and economic barrier against oral diseases at the whole society level and could meet the goals of the Healthy China 2030 plan, the 13th five-year plan, and the Chronic Diseases Program.

To promote a primordial barrier against oral disease, the government needs to correctly handle its relationship between the market. Unnecessary government interventions in oral health care services should be reduced. The government can also guide financial institutions to increase financial support for the oral health industry, with a particular interest on the early diagnosis, early treatment and early rehabilitation of oral diseases. The government can also play a positive role in the creation of a functional dental insurance system appropriate to the socioeconomic status of the country. The current basic medical insurance in China only covers a small portion of dental care expenditures, and over 85% of total dental costs are paid out of pocket^27^. The economic burden of dental treatment restricts people’s access to adequate dental care^28^. The government should encourage the introduction of commercial dental insurance as a complement to the current basic medical insurance and should establish a comprehensive insurance system that acknowledges the equity of basic dental care needs and meets specialized demands for advanced dental care. In addition, the complementary oral insurance policy should not only cover expenditures for disease treatment; more importantly, it should cover the preventive services.

### Common risk factor strategy for the management of oral diseases

Oral health and oral health care practice are intrinsically linked with many other fields of overall health. However, dentistry has long been recognized as a specialty separate from general medicine. The separate identity of dentistry has led to a low priority and a neglect of oral health in overall health agendas. In China, many traditional oral health promotion programs are independent from general health promotion programs, resulting in insufficient social, political and economic attention on oral health. Hence, integrating oral health into overall health strategies and practices is imperative for the promotion of oral health and could even benefit the promotion of general health, particularly in the fight against NCDs.

Different diseases have common risk factors, and it is a waste of resources to address them separately. A more economic and effective approach could be the promotion of public health by controlling the common risk factors of various diseases. Common risk factor strategy can not only benefit high risk groups but also reduce inequity by promoting and improving health conditions for all population^29^. WHO passed a resolution in 2000 at the 31st World Health Assembly stressing the importance of prioritizing risk factors for NCDs that are related to life habits. Oral diseases, cardiovascular diseases, malignant tumors, diabetes and chronic obstructive lung diseases have common risk factors^30^. The Chronic Diseases Program recently released by the state council of China stressed the importance of the common risk factor strategy for the management of NCDs. The integration of oral health into NCD management will attract more attention to the promotion of oral health, and better management of oral diseases in turn can benefit the prevention and treatment of NCDs with shared risk factors. A good example is that oral health professionals and their representatives participated actively in phase-down amalgam use, as proposed by the Minamata Treaty on mercury, which will not only benefit oral health but will also have fundamental influence on the overall health of humans. More importantly, recent studies have shown that better control of oral diseases, particularly periodontitis, could benefit the blood sugar control of diabetic patients and improve the cognitive improvement of patients with Alzheimer’s^17,31^, further underlining the importance of integrating oral health care practice in the management of NCDs with shared risk factors.

Of note, as China’s aging population is becoming a greater socioeconomic problem, a cost-effective common risk factor strategy with extra efforts for elderly people should be put on the agenda. At the same time, policies need to be translated into tangible actions through the coordination and reconciliation of multiple related government departments, giving everyone equitable access to effective prevention and appropriate care.

### Strategic consultations on oral health promotion

Strategic consultations with academic leaders and organizations are indispensable for better translation of health policy into tangible actions. Entrusted by the relevant ministries and commissions, the Chinese Academy of Engineering (CAE) offers consultancy to the government on major programs, planning, guidelines, and policies. Recently, a strategic analysis project, *The oral health management of China in 2035*, led by the academy member professor Zhiyuan Zhang and jointly performed by Shanghai Jiaotong University, Sichuan University and Harbin Medical University using a multicenter approach, was launched. By comparatively analyzing international trends in oral health promotion, this project aims to identify the advantages and disadvantages of current oral health management measures in China and to identify frontiers for the competitive development of oral health in China. Data obtained from the strategic analysis highlighted the need to integrate oral health into overall health. Acceleration of the research and development of precision medicine on oral cancer, as well as the establishment of a sophisticated domestic manufacturing chain of dental equipment and materials, have also been proposed based on the strategic analyses.

The Chinese Stomatological Association (CSA), which is a national academic nonprofit organization of dental professionals and social organizations, functions as a critical link and bridge between the government and the scientific/clinical/industrial communities of stomatology in China. The CSA and its specific committees are capable of uniting the stomatological professions to promote the prosperity, development and innovation of the science and technology of stomatology and to promote the oral and general health of all people in China. Recently, the CSA established a national “love teeth day campaign 2017” slogan as *oral health, human health*, which has attracted huge public recognition of oral health. The CSA is committed to the development of China’s 80/20 strategy for oral health, which will take positive actions on the implementation of whole lifespan oral health promotion to keep 20 or more teeth by the age of 80, and encourage collaboration between local health authorities and subordinate organizations to promote nationwide oral public health. Encouragingly, data obtained from the 4^th^ national epidemic survey on oral health status in China show that oral health promotion has attracted increased attention from the public. Approximately 60% of citizens have basic knowledge regarding oral health, and 84.9% of citizens have a positive attitude toward oral health promotion^20^.

### Standardized residency training for oral health professionals

It is well recognized that the realization of a healthy China also depends on well-educated health professionals who have the clinical, ethical, and human competencies necessary to provide quality services. The current health inequity in China is due less to the shortage of doctors but more to the abundant yet poorly trained health professionals, particularly in underdeveloped rural areas. To standardize the quality of Chinese doctors, the National Health and Family Planning Commission of China, along with six other government ministries, jointly launched the Standardized Residency Training (SRT) program in 2013. The SRT are is to be compulsory nationally for all practicing doctors by 2020^32^. A “5 + 3” track has been proposed, consisting of 5 years of undergraduate medical/stomatological studies (leading to a bachelor degree in medicine/stomatology) followed by additional 3 years of SRT in one of 36 specialties (27 for medicine, seven for stomatology, and two for traditional Chinese medicine)^33^. The SRT program in China shows the government’s determination and commitment to achieving high-quality national health care before 2020. Although the specific curricula for the 3-year SRT in stomatology may vary slightly among different dental schools and hospitals, the ultimate goal is to cultivate general dentists who is competent to provide quality dental services that meet the basic requirements of the Healthy China 2030 plan and its relevant policies. In addition to standardized clinical training in the different specialties in stomatology, a competent dentist through the 3-year SRT is expected to promote oral health by educating the public to cultivate good oral health habits through community and clinical practices. A competent dental care provider should also have good professional ethics and communication skills to appropriately handle relationships with patients, should have critical thinking skills and self-motivation for continued education and post training, and should be familiar with regulations on good dental practice.

### Development of a domestic industrial chain for dental equipment and related supplies

China is currently the second largest market in the world for medical devices. Sales of medical devices in China reached RMB 200 billion in 2013, and medical devices represent one of the fastest growing markets compared with other sectors of the country’s economy^34^. Currently, the market share in China strongly favors foreign companies. It is estimated that approximately 74% of China’s medical device market comes from foreign-owned entities. The percentage of imported products in dentistry is even higher. To tackle this problem, the NHFPC has released a series of policies that favor domestic manufacturers of medical/dental devices. The NHFPC strongly advocates that health organizations, particularly tier-3 top level hospitals, use domestic medical devices and related products. As the public hospital tenders grow broader in scope and as domestically manufactured medical/dental devices and related consumables are specifically recommended, the NHFPC believes that these policies could “effectively control unreasonable increases in the cost of medical care and reduce the burden on patients.”

The NHFPC’s move provides a good opportunity for the development of domestic dental device manufacturers in China. However, we should realize that China still relies heavily on foreign imports for dental supplies and devices. This is particularly the case for high-tech, high-end, and high-price items, for which Germany, the US and Japan are major suppliers. Although there are several thousand local manufacturers, most of these are makers of low-tech products, such as dental handpieces, LED light curing units and dental chairs, etc. In contrast, the majority of high-tech equipment is imported, including cone beam CTs, chair-side CAD/CAM machines, dental ultrasound equipment and dental lasers, as well as high-price consumables such as dental implants, orthodontic appliances, biological bone substitutes and bio-ceramics. Imports of mid-to-high end products, such as dental microscopes, Ni–Ti endo rotary systems and apex locators, have decreased in recent years, but this has largely been due to joint ventures or outsourcing by foreign companies. Currently, many domestic manufacturers are unable or unwilling to make the investments necessary to break the entry barrier for high-end dental products. It is simply easier for domestic companies to copy foreign products, which has led to an upsurge in intellectual property-related issues in recent years. The discrepancy between foreign manufacturers and local companies necessitates the development of a domestic dental industry within the framework of the Healthy China 2030 plan, and this will require great innovation, continuous research and development investment from both the government and entrepreneurs, close collaborations between enterprise and academic institutes/universities, and well-planned marketing. As the prevention-oriented Healthy China 2030 plan is implemented at the national level, an industrial chain focusing on the early diagnosis, early treatment, and early rehabilitation of oral diseases will definitely benefit, and economic and effective public oral health are anticipated.

### Revitalization of traditional Chinese medicine

As prevention-oriented strategies have been highlighted in the recently released healthy policies of the Chinese government, the demands for preventive oral pharmaceuticals and dental health products rather than surgical dental procedures for established diseases are increasing. Traditional Chinese medicine (TCM), which has been developed for more than 2 000 years, has attracted increasing attention recently, particular due to its comparable efficacy and reduced side-effects for the treatment of NCDs^35^. Although standard criteria for post-treatment assessment are not available and the exact of TCM mechanisms are still unclear, TCM has shown effectiveness in the treatment of oral diseases, including dental caries, periodontitis, recurrent aphthous stomatitis, oral lichen planus, leukoplakia, and Sjogren’s syndrome, etc.^36–41^. Researchers from the West China School of Stomatology Sichuan University have identified a series of active compounds from TCM, such as *Nidus vespae*, *Ginkgo biloba*, *Galla chinensis*, *Camellia sinensis*, and *Magnolia officinalis*, that demonstrate comparable effectiveness for the prevention of oral infectious diseases^36–40,42–46^. Clearly, there is great potential for the discovery of pharmaceutical compounds against oral diseases through TCM. However, although multitudes of studies have revealed the beneficial effects of TCM, we still face quite a few challenges to realizing a modernized TCM for oral diseases. The efficacy of many TCM treatments for oral diseases is no better than that of conventional pharmaceuticals, and additional well-controlled studies are still needed to determine the optimal TCM practices for the prevention and treatment of oral diseases. In addition, the extract mode of action and molecular mechanisms of TCM warrant further investigation, and the clinical effects, stability and optimal doses of TCM for the prevention and treatment of oral diseases still need clinical validation.

The China National Health and Wellness Conference 2016 underlined the need to revitalize TCM, particularly to achieve a creative transformation of TCM^1^. This indicates the government’s determination to modernize TCM and to promote a complementary and coordinated development of both TCM and Western medicine. Specific measures to revitalize TCM in dentistry include but are not restricted to promoting the use of TCM for the prevention and treatment of oral diseases, improving the infrastructure of TCM-related pharmaceuticals and the service level of TCM, and supporting the research and development of TCM-related oral health products.

### Dental health promotion and the Belt and Road initiative

The Belt and Road Initiative is a development strategy proposed by Chinese president Xi Jinping that focuses on connectivity and cooperation between China and other Eurasian counties, primarily the land-based Silk Road Economic Belt and the Maritime Silk Road. Although the primary aim of the Belt and Road Initiative relates to multilateral trade, it also provides a platform for bilateral and multilateral regional cooperation to combat common diseases and achieve universal health coverage and equal access to health care^47^.

Recently, *the Belt and Road High Level Meeting for Health Cooperation: Towards a Health Silk Road* was held in Beijing^48^. The general director of WHO as well as more than 20 ministers and deputy ministers of health attended the meeting. The meeting looked at ways to promote collaboration among the Belt and Road countries, with particular emphasis on collaboration in innovation and technology, vaccine safety, nutrition, maternal and child health and human resources for health. Relative to its Belt and Road partners, dentistry in China is well-established; China has a long history of modern dental education and relatively sophisticated domestic manufacturing. To accelerate China’s contributions to global health, Chinese universities and academic societies should proactively strengthen partnerships not only with institutions in developed countries but also with those in low-income and middle-income developing countries. The CSA has been committed to providing medical assistance to the Belt and Road partners and to engaging in regional cooperation in the management of chronic oral diseases, particularly dental caries and periodontitis. The CSA has also agreed to increase regional cooperation for clinical training, academic research and technology development through establishing joint dental schools/hospitals, research centers, and clinical translation centers. In addition, in 2016, the West China School of Stomatology Sichuan University founded the Asian Dental Center, which aims to integrate optimal resources to promote clinical and academic cooperation between dental schools in China and its Belt and Road partners. In parallel, the Guangxi Medical University is building an independent dental school to meet the increasing need for dental professionals with standardized training from members of the Association of Southeast Asian Nations (ASEAN). These ongoing actions demonstrate concerted efforts to fight chronic oral diseases, such as dental caries and periodontal diseases, and can advance health equity and development both nationally and globally.

### Summary

The current social and economic transformation in China, which includes improved life expectancy, “super-speed” aging, mass urbanization, and the abrogation of the one-child policy, has made health promotion the first priority for sustainable economic and social development of China. Since 2016, the Chinese government has released a series of health-related policies underlining the importance of oral health for the economic and social development of China. To fulfill a larger goal for oral health in 2030, it is necessary to implement primordial prevention against oral diseases, to integrate oral health into the promotion of overall health and to manage oral diseases with other NCDs with shared risk factors. In addition, establishing national quality standards for clinical residency, reforming the domestic manufacturing of dental equipment and materials and revitalizing the use of TCM for the prevention and treatment of oral diseases should be part of the agenda. Oral health promotion is a global issue that demands international collaboration, which should also be in accordance with the nation’s Belt and Road Initiative. The government, oral health professionals and domestic enterprises should seize this important opportunity to achieve the ultimate goal of a Healthy China and ensure the oral health of the nation.

## References

[1] Xinhuanet. National Health and Wellness Conference. http://www.xinhuanetcom/health/zt/2016JK20/ (2017).

[2] The State Coucil of China. Healthy China 2030 Plan Blueprint. http://www.govcn/zhengce/2016-10/25/content_5124174htm (2017).

[3] WHO. Global status report on noncommunicable diseases 2014. (2014).

[4] Kassebaum NJ (2017). Global, Regional, and National Prevalence, Incidence, and Disability-Adjusted Life Years for Oral Conditions for 195 Countries, 1990–2015: a systematic analysis for the global burden of diseases, injuries, and risk factors. J. Dent. Res..

[5] Marcenes W (2013). Global burden of oral conditions in 1990-2010: a systematic analysis. J. Dent. Res..

[6] GBD 2016 Disease and Injury Incidence and Prevalence Collaborators. Global, regional, and national incidence, prevalence, and years lived with disability for 328 diseases and injuries for 195 countries, 1990-2016: a systematic analysis for the Global Burden of Disease Study 2016. *Lancet* **390**, 1211–1259 (2017). 10.1016/S0140-6736(17)32154-2 PMC5605509 28919117

[7] Listl S, Galloway J, Mossey PA, Marcenes W (2015). Global economic impact of dental diseases. J. Dent. Res..

[8] Kassebaum NJ (2015). Global burden of untreated caries: a systematic review and metaregression. J. Dent. Res..

[9] Barton MK (2017). Evidence accumulates indicating periodontal disease as a risk factor for colorectal cancer or lymphoma. Ca. Cancer J. Clin..

[10] Pucar A (2007). Correlation between atherosclerosis and periodontal putative pathogenic bacterial infections in coronary and internal mammary arteries. J. Periodontol..

[11] Offenbacher S (1996). Periodontal infection as a possible risk factor for preterm low birth weight. J. Periodontol..

[12] Bartold PM, Marshall RI, Haynes DR (2005). Periodontitis and rheumatoid arthritis: a review. J. Periodontol..

[13] Poole S, Singhrao SK, Kesavalu L, Curtis MA, Crean S (2013). Determining the presence of periodontopathic virulence factors in short-term postmortem Alzheimer’s disease brain tissue. J. Alzheimer’s Dis..

[14] Momen-Heravi F (2017). Periodontal disease, tooth loss and colorectal cancer risk: results from the nurses’ health study. Int. J. Cancer.

[15] Abed J (2016). Fap2 mediates fusobacterium nucleatum colorectal adenocarcinoma enrichment by binding to tumor-expressed Gal-GalNAc. Cell. Host. Microbe.

[16] Singhal S (2011). The role of oral hygiene in inflammatory bowel disease. Dig. Dis. Sci..

[17] Lalla E, Papapanou PN (2011). Diabetes mellitus and periodontitis: a tale of two common interrelated diseases. Nat. Rev. Endocrinol..

[18] Chapple IL, Genco R (2013). Diabetes and periodontal diseases: consensus report of the Joint EFP/AAP Workshop on Periodontitis and Systemic Diseases. J. Periodontol..

[19] Hajishengallis G (2015). Periodontitis: from microbial immune subversion to systemic inflammation. Nat. Rev. Immunol..

[20] National Health and Family Planning Commission. Press Conference on the results obtained from the 4th national oral health epidemiology survey. http://www.nhfpcgovcn/zhuz/xwfb/201709/9b4d4a4ec1c54723820dbaedf97a6d26shtml (2017).

[21] The State Council of China. The blueprint of health plan during the 13th five-year plan period. http://www.govcn/zhengce/content/2017-01/10/content_5158488htm (2017).

[22] The general office of National Health and Family Planning Commission. The Regulation of National Demonstration Area for Comprehensive Prevention and Control of Chronic and Non-Communicable Disease. http://www.nhfpcgovcn/jkj/s5878/201611/6d55c194a965460b9bc7ee9cb5cb4592shtml (2017).

[23] The General Office of State Council of China. National Program for Chronic Disease Control and Prevention (2017–2025). http://www.govcn/zhengce/content/2017-02/14/content_5167886htm (2017).

[24] Liu XN, Gao XJ, Guo CB, Yu GY (2017). Focus on the recent state policy for oral health in China. Chinese J. Stomatol..

[25] Rydin Y (2012). Shaping cities for health: complexity and the planning of urban environments in the 21st century. Lancet.

[26] Flynn BC (1996). Healthy Cities: toward worldwide health promotion. Annu. Rev. Public. Health.

[27] Hu DY, Hong X, Li X (2011). Oral health in China--trends and challenges. Int. J. Oral. Sci..

[28] Liu J, Zhang SS, Zheng SG, Xu T, Si Y (2016). Oral health status and oral health care model in China. Chin. J. Dent. Res..

[29] Sheiham A, Watt RG (2000). The common risk factor approach: a rational basis for promoting oral health. Community Dent. Oral. Epidemiol..

[30] Petersen PE (2004). Continuous improvement of oral health in the 21st century: the approach of the WHO Global Oral Health Programme. Chinese J. Stomatol..

[31] Rolim Tde S (2014). Evaluation of patients with Alzheimer’s disease before and after dental treatment. Arq. Neuropsiquiatr..

[32] National Health and Family Planning Commission, State Commission Office for Public Sector Reform (SCOPSR), National Development and Reform Commission, Ministry of Education, Ministry of Finance, Ministry of Human Resources and Social Security, State Administration of Traditional Chinese Medicine. Guiding opinions on establishing the standardised residency training system. 2013. http://www.mohgovcn/qjjys/s3593/201401/032c8cdf2eb64a369cca4f9b76e8b059shtml (2017).

[33] National Health and Family Planning Commission. Regulations on SRT management (trial). 2014. http://www.mohgovcn/qjjys/s3593/201408/6281beb3830c42c4a0d2319a2668050eshtml (2017).

[34] Torsekar, M. P. U.S. Medical Devices and China’s Market: Opportunities and Obstacles. https://www.usitc.gov/publications/332/id-036workingpaperusmedicaldevicesfinalss.pdf (2017).

[35] Zheng LW, Hua H, Cheung LK (2011). Traditional Chinese medicine and oral diseases: today and tomorrow. Oral. Dis..

[36] Cheng L, Li J, He L, Zhou X (2015). Natural products and caries prevention. Caries Res..

[37] Huang XL, Liu MD, Li JY, Zhou XD, ten Cate JM (2012). Chemical composition of Galla chinensis extract and the effect of its main component(s) on the prevention of enamel demineralization in vitro. Int. J. Oral. Sci..

[38] Cheng L, Li J, Hao Y, Zhou X (2010). Effect of compounds of Galla chinensis on remineralization of enamel surface in vitro. Arch. Oral. Biol..

[39] Xiao J (2006). Effects of Nidus Vespae extract and chemical fractions on the growth and acidogenicity of oral microorganisms. Arch. Oral. Biol..

[40] Xu X, Zhou XD, Wu CD (2011). The tea catechin epigallocatechin gallate suppresses cariogenic virulence factors of Streptococcus mutans. Antimicrob. Agents Chemother..

[41] Li CL, Huang HL, Wang WC, Hua H (2016). Efficacy and safety of topical herbal medicine treatment on recurrent aphthous stomatitis: a systemic review. Drug Des. Dev. Ther..

[42] He J (2013). Effects of ginkgoneolic acid on the growth, acidogenicity, adherence, and biofilm of Streptococcus mutans in vitro. Folia Microbiol..

[43] Zou L (2008). Effect of Galla chinensis extract and chemical fractions on demineralization of bovine enamel in vitro. J. Dent..

[44] Cheng L, Li J, Hao Y, Zhou X (2008). Effect of compounds of Galla chinensis and their combined effects with fluoride on remineralization of initial enamel lesion in vitro. J. Dent..

[45] Xiao J (2007). Effects of Nidus Vespae extract and chemical fractions on glucosyltransferases, adherence and biofilm formation of Streptococcus mutans. Arch. Oral. Biol..

[46] Chu JP, Li JY, Hao YQ, Zhou XD (2007). Effect of compounds of Galla chinensis on remineralisation of initial enamel carious lesions in vitro. J. Dent..

[47] National Health and Family Planning Commission. Progress in Health Silk Road of the Belt and Road Initiative. http://www.nhfpcgovcn/zhuz/xwfb/201705/862119c3357442a9ae66ce998da292dashtml (2017).

[48] Chinese Hospital Association. Belt and Road high level meeting for health cooperation: towards a health silk road. http://wwwchaorgcn/plus/viewphp?aid=15402 (2017).

